# Humoral Immune Response of BNT162b2 and CoronaVac Vaccinations in Hemodialysis Patients: A Multicenter Prospective Cohort

**DOI:** 10.3390/vaccines10091542

**Published:** 2022-09-16

**Authors:** Rene Clavero, Alfredo Parra-Lucares, Gabriel Méndez-Valdés, Eduardo Villa, Karin Bravo, Evelyn Mondaca, Josseline Aranda, Rose Brignardello, Cynthia Gajardo, Angelica Ordenes, Evelyn Colombo, Jessica Tapia, Andoni Etcheverry, José Zúñiga, Luis Toro

**Affiliations:** 1 Fuerza de Trabajo Anti-COVID-19 (FUTAC Team), Sociedad Chilena de Nefrología, Santiago 7500781, Chile; 2 Hospital Gustavo Fricke, Viña del Mar 2570017, Chile; 3 Centro Renal SpA, Valparaíso 2361843, Chile; 4 Division of Critical Care Medicine, Department of Medicine, Hospital Clinico Universidad de Chile, Santiago 8380456, Chile; 5 MD PhD Degree Program, Faculty of Medicine, Universidad de Chile, Santiago 8380456, Chile; 6 School of Medicine, Faculty of Medicine, Universidad de Chile, Santiago 8380456, Chile; 7 Division of Nephrology, Department of Medicine, Hospital Clinico Universidad de Chile, Santiago 8380456, Chile; 8 Centro de Investigación Clínica Avanzada, Hospital Clínico Universidad de Chile, Santiago 8380456, Chile; 9 Critical Care Center, Clinica Las Condes, Santiago 7591047, Chile

**Keywords:** renal dialysis, hemodialysis, COVID-19, SARS-CoV-2, immune response, antibody

## Abstract

The CoronaVac vaccine is the most used anti-SARS-CoV-2 vaccine worldwide. Previous data indicate that this vaccine produces a lower immune response than RNA vaccines such as BNT162b2. End-stage renal disease (ESRD) patients have an increased rate of COVID-19 and a reduced immune response to vaccinations. Currently, there is little data on this population’s immune response induced by CoronaVac. Methods: This study involved a prospective cohort of ESRD patients in chronic hemodialysis who received a two-dose immunization scheme of either CoronaVac (Sinovac Biotech) or BNT162b2 vaccines (Pfizer-BioNTech). We measured the plasma levels of anti-SARS-CoV-2 IgG antibodies. We determined antibody titers before immunization, 2 and 4 months after two doses, plus 4 months after a booster dose. Results: We evaluated 208 patients in three hemodialysis centers. The mean age was 62.6 ± 15.6 years, of whom 91 were female (41.75%). Eighty-one patients (38.94%) received the BNT162b2 vaccine and 127 (61.06%) received the CoronaVac vaccine. Patients who received the BNT162b2 vaccine had a higher humoral response compared to those who received the CoronaVac vaccine (4 months after the second dose: BNT162b2: 88.89%, CoronaVac: 51.97%, *p* < 0.001; 4 months after the booster: BNT162b2: 98.77%, CoronaVac: 86.61%, *p* < 0.001). Conclusions: Our results suggest that the CoronaVac vaccine induced a lower humoral response than the BNT162b2 vaccine in ESRD patients on hemodialysis.

## 1. Introduction

The COVID-19 pandemic has had a significant global impact over the past two years, with over 550,000,000 people infected and over 6,300,000 deaths as of July 2022 [1]. End-stage renal disease (ESRD) patients in renal replacement therapy, including hemodialysis, peritoneal dialysis, and kidney transplants, are a vulnerable population with a higher infection rate and adverse outcomes, including hospitalizations and mortality, compared to the general population [2,3,4].

Given the impact of the pandemic, significant international efforts have been made to design anti-SARS-CoV-2 vaccines and distribute them worldwide. More than ten vaccines have proven effective in clinical studies, primarily in the general population [5,6]. In the general population, these vaccines induce an anti-SARS-CoV-2 immune response, both in cellular and humoral responses [7,8,9]. This immune response is associated with a reduction in COVID-19 infection and a decrease in adverse clinical outcomes, including hospitalization and death [5,7,8]. 

Current data suggest that ESRD patients develop a lower immune response compared to the general population. Hemodialysis patients vaccinated with the BNT162b2 vaccine (Pfizer-BioNTech) induced anti-SARS-CoV-2 antibodies, although at lower concentrations than healthy volunteers [10]. In addition, these patients have an earlier decline in anti-SARS-CoV-2 antibody titers compared to the general population [11]. 

In developed countries, the most used vaccines have been RNA vaccines such as BNT162b2 (Pfizer-BioNTech, Pfizer, New York, NY, USA, and BioNTech, Mainz, Germany), ChAdOx1 nCoV-19 (Oxford-AstraZeneca, University of Oxford, Oxford, UK, and AstraZeneca, Cambridge, UK), and mRNA-1273 (Moderna, Cambridge, MA, USA). However, the most used vaccine worldwide is the CoronaVac vaccine (Sinovac Biotech, Beijing, China), an inactivated whole-virion vaccine, especially in low- and middle-income countries, with almost half of the total doses delivered globally [12]. This is mainly due to its relatively lower cost than the nucleoside-modified RNA vaccines. All these vaccines have demonstrated a reduction in COVID-19 and adverse outcomes, including hospitalizations and deaths [5,7,8]. However, when comparing efficacy in the general population, current data indicates that CoronaVac induces a lower immune response and reduction in clinical outcomes compared to RNA vaccines [13,14,15].

Currently, there are little data that compare the immune responses of CoronaVac and RNA vaccines in ESRD patients on hemodialysis, including both humoral and immune response and epidemiological studies. A recent study by our group suggests that both CoronaVac and BNT162b2 reduce the incidence of COVID-19 and mortality in ESRD patients on hemodialysis. However, the clinical efficacy of BNT162b2 is significantly higher than CoronaVac [16]. 

The objective of this study was to evaluate the effects of the CoronaVac and BNT162b2 vaccines in inducing a humoral immune response against SARS-CoV-2 in a multicenter prospective cohort of ESRD patients on hemodialysis. We compared patients who received an initial vaccination schedule using a two-dose vaccination with CoronaVac and BNT162b2. Also, we evaluated the effect of a third dose (booster dose) on the humoral immune response. 

Our results show that both CoronaVac and BNT162b2 increase titers of anti-SARS-CoV-2 antibodies in these patients. However, the increase induced by BNT162b2 was superior compared to CoronaVac. This difference between the groups was still detected after the booster doses. These results, similar to our data on clinical outcomes, support the realization of a national vaccination campaign aimed at this population, with priority use of RNA vaccines to induce an appropriate immune response and potentially prevent the development of adverse outcomes in this high-risk group.

## 2. Materials and Methods

### 2.1. Study Design

The HDVAC-Ab study, conducted by the FUTAC Team of the Chilean Society of Nephrology, involved a multicenter prospective cohort of patients on chronic hemodialysis in Chile and was conducted in 2021–2022 to determine the production of the humoral immune response after anti-SARS-CoV-2 vaccination. Baseline demographic, laboratory, and clinical data were collected from patients. The evaluation of the humoral immune response was determined by measurements of IgG antibodies against the S1-spike protein of the SARS-CoV-2 virus. Measurements were performed before immunization, after a 2-dose vaccination schedule, and after a third dose (“booster”). This study was approved by the Institutional Ethics Committee.

### 2.2. Chilean Immunization Program

On 2 February 2021, a national anti-SARS-CoV-2 vaccination campaign began in Chile, which included patients with end-stage renal disease (hemodialysis, peritoneal dialysis, and kidney transplant) within the priority vaccination group, regardless of age. The campaign included the use of the inactivated virus anti-SARS-CoV-2 vaccine CoronaVac (Sinovac Biotech) and the messenger RNA vaccine BNT162b2 (Pfizer-BioNTech). The campaign included the administration of two doses of the vaccine to each patient (CoronaVac or BNT162b2) separated by four weeks. The decision about which vaccine would be administered was based on the onsite availability of the vaccines; however, the same vaccine was administered for the two doses. The vaccine administration was funded by Chile’s government and was free for patients. In late 2021, published data, including the results of our group [16], suggested a superior efficacy of BNT162b2 versus CoronaVac to prevent SARS-CoV-2 infection, hospitalization, and deaths. Using this information, the FUTAC Team and the Chilean Ministry of Health modified the recommendations for vaccinations, where ESRD patients were given priority for the BNT162b2 vaccine for the booster doses. Therefore, more than 98% of ESRD patients who had booster doses received the BNT162b2 vaccine.

### 2.3. Inclusion and Exclusion Criteria

We included patients older than 18 years with a diagnosis of end-stage renal disease on chronic hemodialysis who received a two-dose vaccination schedule of BNT162b2 or CoronaVac plus a third booster dose (BNT162b2 in all patients). We excluded patients with a medical contraindication to the vaccination, patients under palliative management, pregnant or lactating women, and patients with incomplete vaccination data. 

### 2.4. Evaluation of Patients

The humoral immune response was evaluated using the VITROS Anti-SARS-CoV-2 IgG Quantitative test^®^ (Ortho Clinical Diagnostics, Raritan, NJ, USA). This product detects human IgG in plasma serum and plasma, which bonds to the SARS-CoV-2 spike protein S1 antigen. This test has Food and Drug Administration (FDA) approval and the CE mark for in vitro diagnostic medical devices (IVDs) and is standardized according to the World Health Organization (WHO)’s standard for measurements of anti-SARS-CoV-2 immunoglobulins. Blood samples were obtained during the hemodialysis session before the initiation of hemodialysis by puncture of arteriovenous fistula or central venous catheter using a 4 mL EDTA tube. Plasma samples were extracted by centrifugation and then used for measurements. Measurements were performed before the first vaccine dose, two months after the second vaccine, four months after the second vaccine, and four months after the booster dose.

Patients were classified as vaccine responders if they presented an antibody titer greater than or equal to 2 U/L. This cutoff was based on the manufacturer’s recommendations and literature data [17]. Non-responder patients were classified if they presented an antibody titer between 0 and 0.99 U/L, and borderline patients were considered if they presented an antibody titer between 1 and 1.99 U/L. Vaccine responsiveness was determined four months after the 2-dose vaccination schedule and four months after the booster dose.

The follow-up of patients was carried out between 2 February 2021 and 31 May 2022. The diagnosis of SARS-CoV-2 was confirmed by a polymerase chain reaction (PCR) test, reported on the Epivigila platform (used by the Chilean Ministry of Health to follow up on COVID-19 patients) [18]. Patients with a COVID-19-related death with laboratory confirmation (PCR test) were analyzed, corresponding to code U07.1 in the International Classification of Diseases, 10th Revision [19].

### 2.5. Statistical Analysis

Discrete variables were expressed as absolute values (percentages) and continuous variables were expressed as arithmetic mean ± standard deviation or median [percentile 25–percentile 75]. For comparisons of baseline data between groups, the chi-square test for discrete variables was used or the Student’s t-test for paired or unpaired groups of continuous variables. To evaluate the predictors of vaccine responsiveness, we performed a multivariate analysis through logistic regression, evaluating the potential demographical, clinical, and laboratory predictors. In this model, we included those variables that presented a *p*-value below 0.10 in the univariate analysis. This multivariate analysis was performed to evaluate vaccine effectiveness after 2 doses and after a booster dose. A *p*-value of less than 5% (*p* < 0.05) was considered statistically significant. The software Stata SE v.15.0 and GraphPad Prism v.8.0 were used for the analyses.

## 3. Results

### 3.1. Baseline Characteristics

A total of 208 patients on chronic renal replacement therapy were recruited for the study. The baseline characteristics of the patients are presented in Table 1. The mean age was 62.6 ± 15.6, of whom 91 were female (43.75%) and 80 had diabetes(38.46%). Eighty-one (38.94%) received the BNT162b2 vaccine and 127 (61.06%) received the CoronaVac vaccine. No statistical differences between the groups were detected in the baseline variables. During follow-up, 29 patients (13.94%) developed COVID-19 and 12 (5.77%) died. Regarding the causes of death, three patients (25%) died of COVID-19 and nine (75%) died of cardiovascular causes unrelated to COVID-19.

### 3.2. Effects of the Two-Dose Vaccination

**Figure 1:**
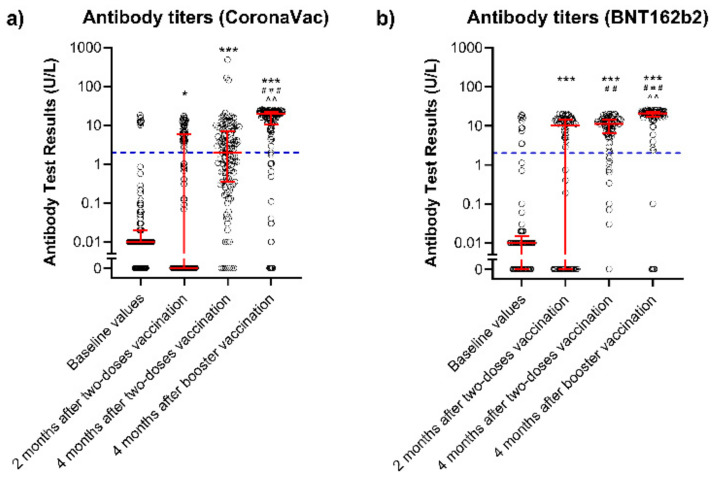
Humoral immune response before and after two vaccine doses and booster doses in end-stage renal disease patients in hemodialysis. Individual patient data of antibody titers before vaccination (baseline values), 2 and 4 months after a two-dose vaccination schedule, and 4 months after a booster dose (scatter dot plot). All patients received the BNT162b2 vaccination as the booster dose, regardless of the previous vaccination (**a**) Patients who received the CoronaVac vaccine and (**b**) patients who received the BNT162b2 vaccine. Blue dotted lines indicate vaccine responsiveness cutoffs (2 U/L). Red lines express median (percentile 25–percentile 75). *** *p* < 0.001, * *p* < 0.05 vs. baseline. ### *p* < 0.001, ## *p* < 0.01 vs. 2 months. ^^ *p* < 0.01 vs. 4 months.

**Figure 2:**
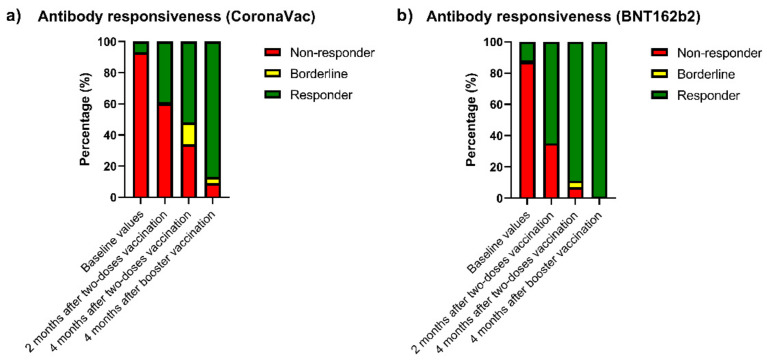
Vaccine responsiveness before and after two vaccine doses and booster doses in end-stage renal disease patients in hemodialysis. Proportion of immune responsiveness before vaccination, 2 and 4 months after a two-dose vaccination schedule, and 4 months after a booster dose. All patients received the BNT162b2 vaccination as the booster dose, regardless of the previous vaccination. Patients were classified as responder (antibody titers ≥ 2 U/L–green bar), borderline (1–1.99 U/L–yellow bar), or non-responder (0–0.99 U/L–red bar). (**a**) Patients who received the CoronaVac vaccine and (**b**) patients who received the BNT162b2 vaccine. Data are expressed as percentages (*p*-values of comparisons are presented in Table 2).

Figure 1 and Table 2 show the temporal evolution of the titers of the anti-SARS-CoV-2 antibodies before and after the two-dose vaccination. Before the vaccination, 19 patients (9.13%) had titers above 2 U/L (11/19 had had previous SARS-CoV-2 infections). After the two-dose vaccination, 102 patients (49.04%) were classified as responders at two months and 138 (66.35%) at four months after the vaccination. When comparing the humoral response of the different vaccines (Figure 2), patients who received the BNT162b2 vaccine had a higher response rate (titers above 2 U/L) compared to those who received the CoronaVac vaccine 2 months after the vaccination (BNT162b2: 65.43%, CoronaVac: 38.58%, *p* = 0.001) and 4 months after the vaccination (BNT162b2: 88.89%, CoronaVac: 51.97%, *p* < 0.001). When comparing responder versus non-responder patients at four months after the two-dose vaccination (66.35% of the total cohort), the responders were younger (58.0 ± 16.1 vs. 71.2 ± 10.6 years, *p* < 0.001) and had higher use of the BNT162b2 vaccine (52.17% vs. 12.86%, *p* < 0.001). Multivariate analysis indicated that the predictors of responsiveness of the humoral immune response were age below 60 years (OR: 1.540 [1.179–2.012], *p* = 0.002) and use of the BNT162b2 vaccine (OR: 4.310 [1.851–10.101], *p* = 0.001, Table 3).

### 3.3. Effects of the Booster Dose on Humoral Immune Response

Eight months after the two-dose vaccination, our patients received a third vaccine dose (booster dose). All patients received the BNT162b2 regardless of the first two doses received, as recommended by the Chilean Ministry of Health and the Chilean Society of Nephrology. The rationale for this decision was the published data [14,15] and our results [16] that indicated that patients with BNT162b2 had higher efficacy in preventing adverse outcomes, including hospitalizations and deaths related to COVID-19.

As detailed in Table 2 and Figure 1, four months after the booster dose, there was a significant increase in the immune responsiveness in the total cohort (66.35% before booster vs. 91.35% after the booster, *p* < 0.001)—patients who received two doses of BNT162b2 (88.89% vs. 98.77%, *p* = 0.037) and patients who received two doses of CoronaVac (51.97% vs. 86.61%, *p* < 0.001). However, the responsiveness in patients who initially received BNT162b2 was significantly higher than in those who received CoronaVac (98.77% vs. 86.61%, *p* = 0.009). In addition, when comparing the responder versus non-responder patients at four months after the booster vaccination, the responders were younger (61.6 ± 15.6 vs. 76.5 ± 6.93 years, *p* < 0.001) and had a higher rate of the BNT162b2 vaccine (42.11% vs. 5.56%, *p* = 0.002).

Finally, multivariate analysis indicated that the predictors of responsiveness of the humoral immune response were age below 60 years (OR: 2.057 [1.340–4.149], *p* = 0.044), and use of the BNT162b2 vaccine for the first two doses (OR: 8.849 [1.166–83.333], *p* = 0.041, Table 4).

## 4. Discussion

### 4.1. Effects of Two-Dose Vaccination on Immune Response in ESRD Patients on Hemodialysis

This multicenter observational study showed that both the BNT162b2 and CoronaVac vaccines induced a humoral immune response, evaluated as an increase in the plasma levels of anti-SARS-CoV-2 antibodies in patients with end-stage renal disease on chronic hemodialysis. In addition, a higher humoral immune response was observed with BNT162b2 compared with CoronaVac after two doses and after the BNT162b2 booster vaccination. This is one of the first studies to compare the humoral immune response of CoronaVac and an RNA vaccine such as BNT162b2 in patients undergoing chronic hemodialysis. To date, most of the studies that have evaluated the effects of anti-SARS-CoV-2 vaccinations in hemodialysis patients (including humoral and cellular immune response and clinical efficacy to prevent COVID-19 and COVID-19-related adverse outcomes) have analyzed RNA vaccines such as BNT162b2 (Pfizer-BioNTech) [20], ChAdOx1 nCoV-19 (Oxford-AstraZeneca) [21], and mRNA-1273 (Moderna) [22]. 

A recent epidemiological study by our group [16] evaluated the clinical efficacy of the BNT162b2 and CoronaVac vaccinations to prevent COVID-19, hospitalizations, and deaths related to COVID-19 in hemodialysis patients (two-dose vaccinations). We found that both vaccines reduced the incidence of COVID-19 and adverse outcomes. However, the clinical efficacy of BNT162b2 was superior compared to CoronaVac. These results concord with this study’s findings on the humoral immune response.

### 4.2. Effects of the Booster Dose

The effect of a booster dose to increase antibody titers has been demonstrated in the general population [15,23] and hemodialysis patients [24]. Although our study only included the BNT162b2 as the booster dose in all patients, our results support the use of the BNT162b2 vaccine as a booster vaccine. In addition, previous data indicated that the use of a heterologous scheme of BNT162b2 after two doses of CoronaVac was associated with increased titers of IgG RBD antibodies and neutralizing antibodies compared to homologous schemes with a booster dose of CoronaVac [14,15,25]. These data were used to justify the national implementation of BNT162b2 as the preferred vaccine for booster doses for ESRD patients on hemodialysis in Chile.

### 4.3. Comparison with Previous Data of the Humoral Response Induced by BNT162b2 and CoronaVac Vaccines in ESRD Patients on Hemodialysis

Comparing our results with previous studies that have analyzed CoronaVac in hemodialysis patients, a Turkish study [26] evaluated the effects of two-dose vaccinations in 50 hemodialysis patients. They detected a humoral immune response between 78 and 86% at 3–6 months after the vaccination, which was a higher rate than our results. These differences may be due to their cohort of patients being younger than those in our group, a variable that is an independent predictor of vaccine responsiveness.

Although this study was not designed to compare the effects of vaccinations on the humoral immune response of the general population and patients on hemodialysis, our results suggest that the effects of vaccination on patients on hemodialysis are lower than those on the general population. Previous studies have reported the presence of a humoral immune response of over 90% after two doses of CoronaVac [27] and nearly 100% after two doses of BNT162b2 [15] in the general population. These results are higher than the immune response found in our group of patients, who had a responsiveness rate of 52% after two doses of CoronaVac and 88% after two doses of BNT162b2. Although the comparison of our results and the literature is limited, it is concordant with previous data on the reduced induction of immune responses after vaccinations in ESRD patients.

### 4.4. Strengths and Limitations of the Study

One of the strengths of this work is the prospective design of our study, which evaluated the patients four times: before vaccination, two months and four months after a two-dose vaccination, and four months after a booster dose. In addition, we included information on the relevant demographic and clinical baseline variables, which have been associated with an impaired humoral response after vaccination, such as age and the presence of diabetes. Also, it evaluated a real-world vaccination schedule, which has been implemented in different locations worldwide. This suggests that our results potentially apply in other locations where these vaccines have been used.

Regarding the limitations of this study, its observational nature has intrinsic biases, as some influencing patient variables are challenging to isolate. For example, non-responsive patients were older than responsive patients after two doses and a booster dose, which may have influenced the differences between the effects of the CoronaVac and BNT162b2 vaccines. The effect of age as a risk factor for impaired immune response has been described in previous studies [28,29,30,31]. To reduce this potential bias, we performed statistical adjustments of the potential confounding variables, including multivariate logistic regression, to evaluate the impact of the vaccination. Also, the baseline characteristics of patients were similar to those vaccinated with both the CoronaVac and BNT162b2 vaccines, suggesting that these demographical and clinical variables had a limited effect on the differences detected between the humoral immune responses to both vaccines.

## 5. Conclusions

The results suggest that COVID-19 vaccinations in patients on chronic hemodialysis are highly effective in inducing a humoral immune response, with greater effects for the BNT162b2 compared to the CoronaVac vaccine, especially after a third booster dose. Our study supports the importance of conducting national vaccination campaigns aimed at this high-risk population to develop an appropriate immune response and prevent the development of adverse outcomes in this vulnerable group.

## Figures and Tables

**Table 1:** Baseline patient characteristics. Data of the total cohort stratified by the vaccine used for the two-dose vaccination (BNT162b2 or CoronaVac) are detailed. Data are expressed as numbers (N) and percentages (%). The *p*-values of BNT162b2 vs. CoronaVac are detailed.

Characteristics	Total Cohort		Patients with BNT162b2 Vaccine		Patients with CoronaVac Vaccine		*p*-Value
	N	%	N	%	N	%	
Total	208	100%	81	38.94%	127	61.06%	-
Sex							
Female	91	43.75%	40	49.38%	51	40.16%	0.191
Male	117	56.25%	41	50.62%	76	59.84%	
Age group							
18–19 years	2	0.96%	1	1.23%	1	0.79%	0.153
20–29 years	7	3.37%	2	2.47%	5	3.94%	
30–39 years	13	6.25%	4	4.94%	9	7.09%	
40–49 years	22	10.58%	10	12.35%	12	9.45%	
50–59 years	34	16.35%	21	25.93%	13	10.24%	
60–69 years	57	27.00%	18	22.22%	39	30.71%	
70–79 years	51	24.52%	18	22.22%	33	25.98%	
≥80 years	22	10.58%	7	8.64%	15	11.81%	
Diabetes							
No	128	61.54%	55	67.90%	73	57.48%	0.132
Yes	80	38.46%	26	32.10%	54	42.50%	
Hypertension							
No	65	31.25%	25	30.86%	40	31.50%	0.924
Yes	143	68.75%	56	69.14%	87	68.50%	
Autoimmune disease							
No	199	95.67%	78	96.30%	121	95.28%	0.724
Yes	9	4.33%	3	3.70%	6	4.72%	
Cancer							
No	198	95.19%	78	96.30%	120	94.49%	0.552
Yes	10	4.81%	3	3.70%	7	5.51%	
Previous SARS-CoV-2 infection							
No	196	94.23%	74	91.36%	122	96.06%	0.156
Yes	12	5.77%	7	8.64%	5	3.94%	

**Table 2:** Humoral immune response of patients before and after anti-SARS-CoV-2 vaccination. Plasma levels of IgG anti-SARS-CoV-2 were measured before vaccination, 2 months and 4 months after a two-dose vaccination, and 4 months after a booster dose. The results of the total cohort stratified by the vaccine used for the two-dose vaccination (BNT162b2 or CoronaVac) are detailed. Data are expressed as numbers (N) and percentages (%). *p*-values of comparisons are detailed.

Characteristics	Total Cohort		*p*-Value vs. Baseline	*p*-Value vs. 4 Months after 2 Doses	Patients with BNT162b2 Vaccine		*p*-Value vs. Baseline	*p*-Value vs. 4 Months after 2 Doses	Patients with CoronaVac Vaccine		*p*-Value vs. Baseline	*p*-Value vs. 4 Months after 2 Doses	*p*-Value BNT162b2 vs. CoronaVac
	N	%			N	%			N	%			
Total	208	100%	n/a		81	38.94%	n/a		127	61.06%	n/a	n/a	n/a
Before anti-SARS-CoV-2 vaccination (U/L)													
Non-responder (0–0.99)	188	90.38%	n/a	<0.001	70	86.42%	n/a	<0.001	118	92.91%	n/a	<0.001	0.192
Borderline (1–1.99)	1	0.48%			1	1.23%			0	0.00%			
Responder (≥2)	19	9.13%			10	12.35%			9	7.09%			
2 months after 2 doses anti-SARS-CoV-2 vaccination (U/L)													
Non-responder (0–0.99)	104	50.00%	<0.001	<0.001	28	34.57%	<0.001	<0.001	76	59.84%	<0.001	<0.001	0.001
Borderline (1–1.99)	2	0.96%			0	0.00%			2	1.57%			
Responder (≥2)	102	49.04%			53	65.43%			49	38.58%			
4 months after 2 doses anti-SARS-CoV-2 vaccination (U/L)													
Non-responder (0–0.99)	49	23.56%	<0.001	n/a	6	7.41%	<0.001	n/a	43	33.86%	<0.001	n/a	<0.001
Borderline (1–1.99)	21	10.10%			3	3.70%			18	14.17%			
Responder (≥2)	138	66.35%			72	88.89%			66	51.97%			
4 months after booster dose anti-SARS-CoV-2 vaccination (U/L)													
Non-responder (0–0.99)	13	6.25%	<0.001	<0.001	1	1.23%	<0.001	0.037	12	9.45%	<0.001	<0.001	0.009
Borderline (1–1.99)	5	4.40%			0	0.00%			5	3.94%			
Responder (≥2)	190	91.35%			80	98.77%			110	86.61%			

**Table 3:** Evaluation of predictors of vaccine responsiveness 4 months after the two-dose vaccination. Univariate and multivariate logistic regression of demographic, clinical, and laboratory parameters of patients. Variables included in the multivariate model were those with a *p*-value below 0.1 in the univariate analysis. Variables considered as predictors of vaccine effectiveness were those with a *p*-value below 0.05 in the multivariate model. Odds ratios and their 95% confidence intervals (95% CI) and *p*-values are detailed.

	Univariate Analysis				Multivariate Analysis			
Variable	Odds Ratio	95% CI		*p*-Value	Odds Ratio	95% CI		*p*-Value
Male	1.399	0.782	2.504	0.258				
Age < 60 years	1.904	1.477	2.457	<0.001	1.540	1.179	2.012	0.002
Diabetes	0.744	0.412	1.343	0.327				
Hypertension	1.493	0.808	2.757	0.200				
Autoimmune disease	1.047	0.254	4.318	0.949				
Cancer	0.640	0.166	2.462	0.516				
Hemodialysis center	1.203	0.824	1.757	0.337				
Vaccine type (BNT162b2)	7.633	3.508	16.667	<0.001	4.310	1.851	10.101	0.001

**Table 4:** Evaluation of predictors of vaccine responsiveness 4 months after the booster vaccination dose. Univariate and multivariate logistic regression of demographic, clinical, and laboratory parameters of patients. Variables included in the multivariate model were those with a *p*-value below 0.1 in the univariate analysis. Variables considered as predictors of vaccine effectiveness were those with a *p-*value below 0.05 in the multivariate model. Odds ratios and their 95% confidence intervals (95% CI) and *p*-values are detailed.

	Univariate Analysis				Multivariate Analysis			
Variable	Odds Ratio	95% CI		*p*-Value	Odds Ratio	95% CI		*p*-Value
Male	1.991	0.663	5.976	0.220				
Age < 60 years	2.352	1.340	4.132	<0.001	2.057	1.340	4.149	0.044
Diabetes	1.095	0.021	3.437	0.303				
Hypertension	1.188	0.381	3.702	0.767				
Autoimmune disease	1.047	0.254	4.318	0.949				
Cancer	0.676	0.079	5.757	0.720				
Hemodialysis center	1.556	0.747	3.239	0.237				
Vaccine type (BNT162b2)	9.090	1.166	71.428	<0.001	8.849	1.166	83.333	0.041

## Data Availability

The data that support the findings of this study are available upon reasonable request from the corresponding author, L.T. The data are not publicly available because they contain information that could compromise the privacy of the research participants and third-party restrictions.
